# Evaluation and Comparison of the Efficacy and Safety of Erbium YAG Laser Along With Normal Saline vs. Its Combination With Stromal Vascular Fraction (SVF) and Platelet‐Rich Plasma (PRP) in the Treatment of Striae Distensae: A Double‐Blind Randomized Clinical Trial

**DOI:** 10.1111/jocd.16757

**Published:** 2025-01-21

**Authors:** Masoumeh Roohaninasab, Zeinab Mahdi, Sona Zare, Abbas Dehghani, Azadeh Goodarzi, Niloufar Najar Nobari, Roya Zeinali, Maryam Nouri, Zahra Ebrahimi, Mohammad Ali Nilforoushzadeh, Elham Behrangi

**Affiliations:** ^1^ Department of Dermatology, Rasool Akram Medical Complex Clinical Research Development Center (RCRDC) School of Medicine, Iran University of Medical Sciences (IUMS) Tehran Iran; ^2^ Skin and Stem Cell Research Center Tehran University of Medical Sciences Tehran Iran; ^3^ Laser Application in Medical Sciences Research Center Shahid Beheshti University of Medical Sciences Tehran Iran; ^4^ Stem Cell and Regenerative Medicine Institute, Sharif University of Technology Tehran Iran; ^5^ Department of Mechanical Engineering Sharif University of Technology Tehran Iran; ^6^ Department of General Medicine Iran University of Medical Sciences Tehran Iran; ^7^ Skin Repair Research Center Jordan Dermatology and Hair Transplantation Center Tehran Iran

**Keywords:** clinical trial, efficacy, Er:YAG, erbium YAG laser, platelet‐rich plasma, PRP, safety, striae distensae, stromal vascular fraction, SVF

## Abstract

**Background:**

There is no definitive solution for the treatment of striae distensae (SD), and effectiveness of each treatment method remains controversial. We aimed to investigate and compare the efficacy of the combination of Erbium YAG (Er:YAG) laser and stromal vascular fraction (SVF), the combination of Er:YAG laser and platelet‐rich plasma (PRP) and Er:YAG laser plus saline in the treatment of SD.

**Materials and Methods:**

In 12 participating patients with at least three lesions (36 lesions in total), each lesion was treated with an Er:YAG laser. SVF was randomly injected intradermally for the first lesion, PRP for the second lesion, and normal saline as placebo for the third lesion. Treatment duration was one session, and the safety and efficacy of the treatment was assessed 3 months later using the following items: evaluation of biometric parameters including corneometery, erythema, melanin, tewametery, color, cutometery, complete thickness, epidermal thickness, dermal thickness, complete density, epidermal density, and dermal density; assessment of patient and physician satisfaction using Likert score; and recording of adverse effects of treatment.

**Results:**

The study involved 12 patients (evaluating 36 lesions in total), predominantly women (83%), with an average age of 39.16 years. The analysis revealed significant improvements in biometric indices, including complete thickness, epidermal thickness, dermal thickness, and R5 cutometer readings across all groups after the intervention. Notably, the SVF and PRP groups showed statistically significant enhancements in dermal and complete density compared to the placebo group. The SVF group demonstrated a significant increase in epidermal density (from 45.95 to 51.19, *p* = 0.001), unlike the PRP and placebo groups which showed nonsignificant changes. Comparing the average changes in biometric factors, the SVF group exhibited significantly greater increases in complete thickness, dermal thickness, complete density, epidermal density, and dermal density than the other groups. Both patient and physician satisfaction scores were highest in the SVF group (*p* = 0.001), with no significant posttreatment complications reported.

**Conclusion:**

Our results showed that the combination of Er:YAG laser and SVF injection could be considered an effective and safe treatment method in the treatment of SD.

**Trial Registration:**

IRCT20200127046282N14

## 1 Introduction

Striae distensae (SD), commonly known as stretch marks are visible and linear atrophic scars resulting from skin damage caused by excessive stretching of the skin. The prevalence of striae distensae is very high. They occur twice as often in women as in men, and they have also been reported in different age groups [1, 2]. There are two forms of SD including striae rubrae and striae albae. The acute stage (striae rubrae) presents as red and flat erythematous scar (swollen in some cases), while the chronic stage (striae albae) is the pale (hypopigmented), wrinkled, and atrophic SD [3]. Histologically, SD are regarded as epidermal atrophy accompanied by alterations in connective tissue [4].

The abdomen, hips, thighs, and breasts are most commonly affected. Striae usually occur at various physiological stages, such as pregnancy (striae gravidarum), growth spurts during puberty, or rapid physical changes such as obesity or weight loss [5]. This complication is also observed in pathological conditions with hypercortisolism including Cushing disease and genetic disorders such as Marfan syndrome [6]. SD can sometimes occur as a side effect of medications such as topical or systemic corticosteroids and antiretroviral protease inhibitors (e.g., indinavir) [7].

Although striae are harmless complications, their impact on the beauty and appearance of the body can be psychologically disturbing to those affected, which has made striae treatment a necessity for many patients. Existing treatments include the use of topical medications such as tretinoin, hyaluronic acid, ascorbic acid, and botanical oils, as well as treatment methods based on procedures such as laser therapy, light therapy, carboxytherapy, radiofrequency, and microdermabrasion [8]. A recent review showed that injection‐based treatments, including PRP, gave the highest rates of complete response for the treatment of SD [9].

The Erbium YAG (Er:YAG) laser is a laser system that operates at a wavelength of 2940 nm (infrared) and its absorption maximum is related to water. Er:YAG laser skin resurfacing involves controlled physical destruction of the skin with two goals: Epithelialization and collagen regeneration. This process results in improvement of skin texture and relief [10, 11]. The 2940‐nm Er laser has been used in SD treatment in various studies, achieving satisfactory results in terms of increasing epidermal and dermal thickness, as well as higher collagen and elastin density [12, 13, 14].

Adipose tissue is rich in types of germ cells that can have regenerative properties. One of the products made from adipose tissue is stromal vascular fraction (SVF). This product is made from autologous adipose tissue containing adipose‐derived stem cells (ADSCs), macrophages, and endothelial progenitor cells, which makes the product more regenerative due to the heterogeneity of these cells. These cells cause angiogenesis, immunomodulation, differentiation, and extracellular matrix secretion in the injected tissue [15, 16, 17].

Platelet‐rich plasma (PRP), which contains endogenous platelets whose concentration is 3–5 times the normal plasma concentration, is produced mainly by centrifugation. Platelets secrete at least seven essential protein growth factors that play a role in the initiation of wound healing. These factors include isomers of platelet‐derived growth factor (PDGF), transforming growth factor (TGF), vascular endothelial growth factor (VEGF), and epithelial growth factor (EGF). Approximately 95% of growth factors are secreted within 1 h of their production and bind to their receptors located on the graft, flap, or injured cells [13]. In the field of aesthetics, PRP is used for facelift, neck lift, breast augmentation, breast reduction, and autologous fat transfer. Due to the effect of PRP in healing and wound repair, stimulating the production of thick collagen bundles, this method has recently been used as a selective or complementary treatment along with laser therapy for skin renewal and rejuvenation [18].

There are a variety of treatment options with varying efficacy for the treatment of SD, from various topical medications to lasers and energy‐based devices have been reported to date. However, a definitive solution for the treatment of this condition has not yet been found, and the efficacy of each treatment method remains controversial, because of the variety in striae types [4, 9, 19]. Considering this, it is very important to have a basic understanding of the different treatment options and to choose the most effective and safe method, as well as to ensure proper patient counseling to optimize the treatment outcome. Due to the lack of studies in this field, in this study we decided to investigate and compare the efficacy and safety of the combination of Er:YAG laser and SVF, the combination of Er:YAG laser and PRP and Er:YAG laser alone in the treatment of SD in the form of a blinded randomized clinical trial.

## 2 Materials and Methods

### 2.1 Patients

This randomized, double‐blind clinical trial was conducted in 12 patients aged 18–60 years with at least three lesions of striae alba (36 lesions in total) who were referred to a dermatology clinic from March to June 2022. Exclusion criteria were the use of a treatment method for the lesion in the previous 6 months, the use of steroids or immunosuppressive drugs, collagen vascular diseases, pregnancy and lactation, and the presence of skin diseases at the treatment site such as infections. Written informed consent was obtained from patients before the procedure to assure them that the therapeutic procedures would not involve complications or impose costs on them.

At baseline, patients' background information was collected and entered into the study checklist.

The study is registered in Iranian Registry.

This study included three intervention groups: Er:YAG laser+normal saline injection (placebo group), Er:YAG laser+SVF injection (SVF group), and Er:YAG laser+PRP injection (PRP group). One dermatologist was assigned to perform the procedure. Three 10 × 10 cm SD lesions in each patient were marked at the baseline. Each lesion was first treated with the Er:YAG laser, and then the lesions were randomly divided into three groups: the control group (normal saline as placebo) and the case groups with SVF injection and PRP injection, using a randomized list. The condition of SD was assessed both qualitatively through clinical imaging and quantitatively through biometric parameters.

### 2.2 Randomization and Blinding

The type of treatment was randomly selected for each lesion from a box of sealed envelopes that contained codes A, B, and C. The treatment was administered to each lesion.

The study was conducted as a double‐blind trial. The injections were performed with similar syringes, and patients did not know in each lesion which type of treatment they were receiving. The physician who evaluated the clinical images and the statistical expert also did not know which treatment method each patient had used.

### 2.3 SVF Cells Isolation

SVF cells preparation [20] was performed by removing 20 cc of fat from the human thigh or abdomen, apart from intervention area, during a surgical procedure, 3 mm incision, and traditional liposuction. The lipoaspirate was first washed thoroughly in phosphate‐buffered saline (PBS) (Miltenyi Biotec) (without antibiotic) (three times) before undergoing enzymatic digestion with collagenase Type II (Worthington Biochemical) to obtain a single cell suspension for 45 min in 37°C incubator. After digestion, the centrifuged (1500 RPM, 10 min) cell pellet (called the Stromal Vascular Fraction (SVF) cells) was resuspended in normal saline before being serially filtered through 100‐μm mesh filter. The cell sample was counted and resuspended in normal saline. The cell sample was counted. Some of the suspension was subjected to flow cytometry (Partec, Görlitz, Germany) for evaluating cell surface markers and cell viability.

### 2.4 PRP Preparation

In this study, PRP was prepared according to the American Association of Blood Banks [21]. For this purpose, PRP was prepared in two stages using light—spin ‘centrifugation (to separate PRP from whole blood) and then heavy—spin’ centrifugation (to separate platelet poor plasma [PPP]). First, the anticoagulant was added using a 20 mL syringe at a ratio of 1–10 (one milliliter of anticoagulant to ten milliliters of patient’ blood sample). The blood sample was then drawn from the patients' internal cubital vein. About 20 mL of whole blood was collected in tubes containing anticoagulant citrate dextrose solution (ACDA). The tubes were then centrifuged at 1500 RPM for 8 min. The whole serum was collected and recentrifuged at 3800 RPM for 8 min. The upper layer (2/3 tube) containing the poor plasma platelet was then discarded and the sediments, which were PRP resuspended in plasma (1/3 tube), were collected in three 1 cc syringes.

### 2.5 Interventions

Twenty minutes before the initiation of the intervention, 2.5% lidocaine/prilocaine cream (Xyla‐P, Tehran Chemie Pharmaceuticals, Iran) under occlusion was applied for local analgesia. The lesions were cleaned with sterile gauze and disinfected using 70% alcohol swabs before the intervention.

The 2940 nm fractional long‐pulsed Er:YAG laser (Lotus II, Laseroptek, Korea) with an energy of 1000 mJ, fluence of 5 J/cm^2^, frequency of 5–10 Hz, spot size of 5 mm, and duration up to 1000 milliseconds with two passes was conducted in all groups.

2–3 cc of injectable normal saline serum was injected intradermally using a 30‐gauge, 1 cc syringe at one‐centimeter intervals in placebo group. 2–3 cc of the prepared SVF solution using a 30‐gauge, 1 cc syringe with varnish were injected intradermally at one‐centimeter intervals in SVF group. The prepared PRP injection method was the same as the SVF and saline injection: 2–3 cc of PRP was injected intradermally using a 30‐gauge, 1 cc syringe at one‐centimeter intervals.

All patients were prescribed to apply betamethasone ointment for three nights, zinc oxide ointment twice a day for a week, avoid sun exposure for a week and moisturize as needed.

### 2.6 Assessment Methods

In all patients, baseline and 3‐month postprocedure evaluations were conducted based on the following measurements:

1. Evaluation of biometric parameters with the VisioFace (Courage + Khazaka, cologne, Germany) including corneometery [22] (in order to measure stratum corneum layer hydration), erythema, melanin, tewametery (in order to measure transepidermal water loss), colorimetery [23], cutometery [24] (in order to measure tissue elasticity including R2: viscoelasticity, R5: pure elasticity, and R7: proportion of the immediate recovery compared with the amplitude after suction), skin ultrasonic imaging [25] (complete thickness, epidermal thickness, dermal thickness, complete density, epidermal density, and dermal density);
2. Determination of physician and patient satisfaction based on Likert [26] scale (no response (1), little (2), somewhat (3), good (4), and excellent (5)) by evaluating before‐after images; and.
3. Finally, the probable complications for each group were recorded.

### 2.7 Data Analysis

Descriptive results were expressed as mean (± standard deviation, SD). To compare continuous variables before and after the intervention, the paired samples *t*‐test was used. To compare the mean change in biometric factors between the different groups, the one‐way test ANOVA with post hoc was used. All data were checked for normality test. The statistical significance level was set at 0.05. All data were analyzed using SPSS, version 22.0, Armonk, NY, USA: IBM Corp. Released 2015.

## 3 Results

### 3.1 Preparation of Cells and Characterization

**FIGURE 1:**
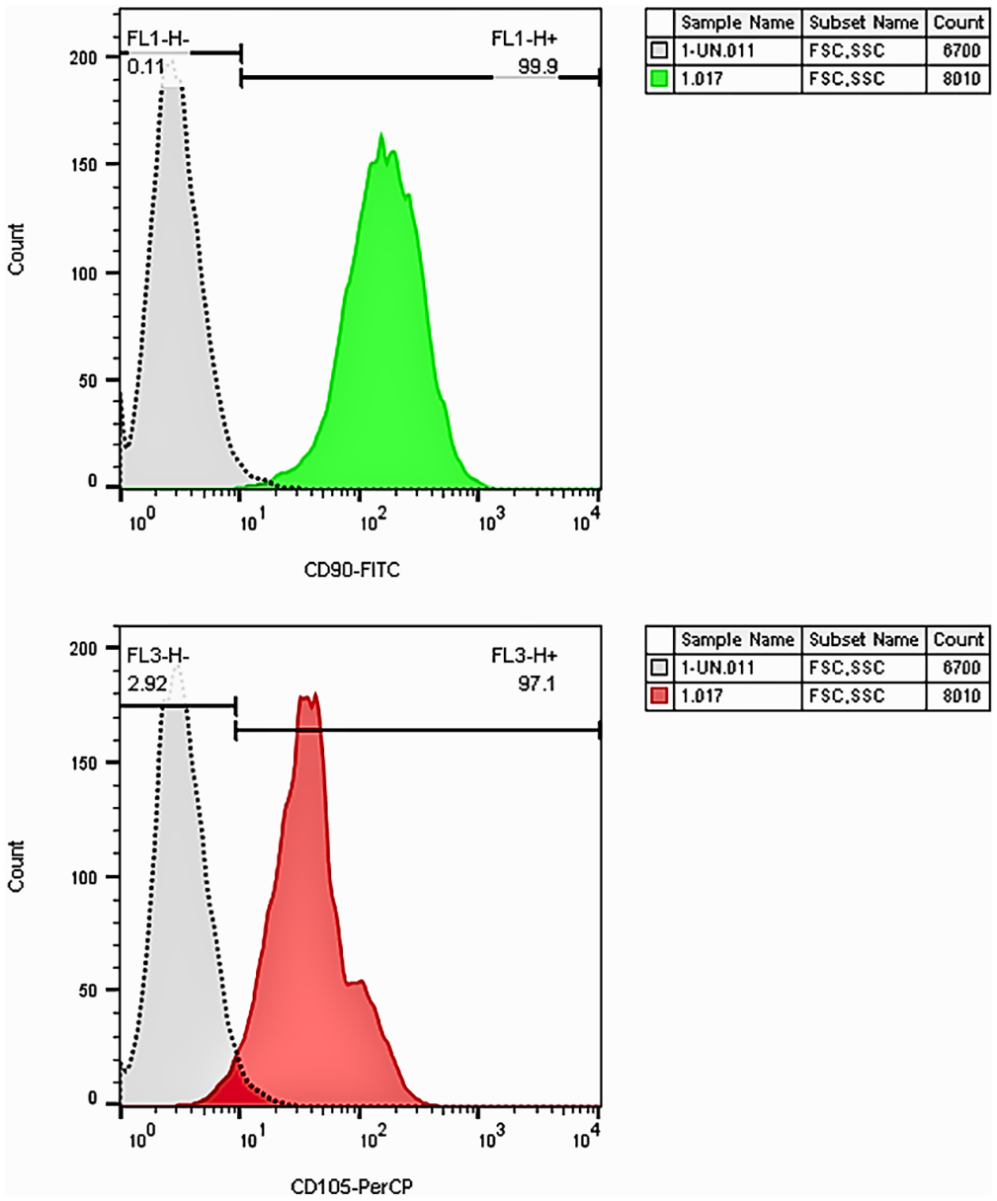
Flow cytometry results in SVF cells.

The mean of yield cell was 5 × 10 [6] cells/mL of fat tissue, and the mean of viability was 91.2%. SVF cells surface markers (CD90 and CD105) and cell viability were evaluated by flow cytometry (Figure 1).

### 3.2 Comparing the Average of Biometric Factors Before and After the Intervention

Of the 12 cases, 10 (83%) were women and the mean age of the patients studied was 39.16 ± 4.66 years, ranging from 28‐ to 47‐year‐old.

Table 1 demonstrates the changes in biometric indices in each group. In comparing the average of biometric factors before and after the intervention, all three groups showed significant improvements in complete thickness, epidermal thickness, and dermal thickness in skin ultrasound indices, as well as in R5 cutometer readings (*p* < 0.05).

**TABLE 1:** Comparing the average of biometric factors before and after the intervention in different groups.

Factor	Before intervention	After intervention	*p*
	Mean ± SD	Mean ± SD	
SVF (*N* = 12)			
Tewameter	9.28 ± 1.99	9.23 **±** 3.05	0.91
Corneometer	33.38 ± 5.30	30.47 **±** 6.64	0.22
Erythema	265.42 ± 82.22	254.61 **±** 45.90	0.45
Melanin	185.33 ± 78.27	176.14 **±** 43.76	0.39
Cutometer R2	0.83 ± 0.10	0.84 **±** 0.04	0.81
Cutometer R5	0.87 ± 0.14	1.01 **±** 0.14	**0.001**
Cutometer R7	0.60 ± 0.10	0.63 **±** 0.03	0.29
Complete thickness (μm)	1012.00 ± 118.22	1198.42 **±** 79.32	**0.001**
Epidermal thickness (μm)	91.50 ± 17.65	108.00 **±** 14.96	**0.001**
Dermal thickness (μm)	920.50 ± 118.09	1090.42 **±** 78.11	**0.001**
Complete density	19.75 ± 5.28	24.00 **±** 4.23	**0.006**
Epidermal density	45.95 ± 5.93	51.19 **±** 2.89	**0.001**
Dermal density	17.03 ± 5.38	21.27 **±** 4.58	**0.007**
PRP (*N* = 12)			
Tewameter	9.25 ± 2.15	8.28 ± 1.69	0.05
Corneometer	34.46 ± 4.57	32.95 ± 4.99	0.47
Erythema	277.31 ± 77.14	286.42 ± 54.46	0.47
Melanin	190.94 ± 81.87	183.50 ± 47.01	0.57
Cutometer R2	0.82 ± 0.07	0.82 ± 0.07	0.64
Cutometer R5	0.87 ± 0.18	0.95 ± 0.23	**0.03**
Cutometer R7	0.58 ± 0.11	0.59 ± 0.12	0.62
Complete thickness (μm)	1022.25 ± 61.51	1104.17 ± 88.14	**0.001**
Epidermal thickness (μm)	94.50 ± 17.01	105.25 ± 15.04	**0.001**
Dermal thickness (μm)	927.75 ± 57.56	998.92 ± 82.25	**0.001**
Complete density	20.01 ± 5.88	23.17 ± 4.04	**0.006**
Epidermal density	46.54 ± 7.93	49.00 ± 3.52	0.15
Dermal density	17.23 ± 5.56	20.42 ± 4.17	**0.004**
Placebo (*N* = 12)			
Tewameter	8.63 ± 2.25	8.85 ± 3.16	0.747
Corneometer	31.03 ± 5.25	29.39 ± 5.44	0.255
Erythema	277.86 ± 97.68	257.86 ± 74.23	0.387
Melanin	177.36 ± 99.38	173.61 ± 75.34	0.783
Cutometer R2	0.83 ± 0.15	0.83 ± 0.12	0.842
Cutometer R5	0.84 ± 0.19	0.97 ± 0.23	**0.029**
Cutometer R7	0.58 ± 0.17	0.60 ± 0.16	0.296
Complete thickness (μm)	972.83 ± 71.88	1046.42 ± 71.05	**0.001**
Epidermal thickness (μm)	90.17 ± 7.51	101.33 ± 10.96	**0.001**
Dermal thickness (μm)	882.67 ± 73.68	945.08 ± 76.49	**0.001**
Complete density	19.11 ± 1.96	19.99 ± 2.54	0.412
Epidermal density	46.79 ± 6.28	47.47 ± 7.21	0.542
Dermal density	16.52 ± 2.59	17.06 ± 2.84	0.652

*Note:* Bold values are statistically significant.

The changes in dermal and complete density were statistically significant in the SVF and PRP groups compared with the placebo group.

There was a significant increase in epidermal density only in the SVF group (increasing from 45.95 ± 5.93 to 51.19 ± 2.89, *p* = 0.001), compared with an increase from 46.54 ± 7.93 to 49.00 ± 3.52 (*p* = 0.15) in the PRP group and from 46.79 ± 6.28 to 47.47 ± 7.21 (*p* = 0.542) in the placebo group.

### 3.3 Comparing the Average Change of Biometric Factors Between Different Groups

The average change of the complete thickness, dermal thickness, complete density, epidermal density, and dermal density indices after the interventions compared with before the interventions, in the SVF group were significantly higher than the other groups (*p* < 0.05). This means that the value of these indices in the SVF group have increased more than in the other two groups (Table 2).

**TABLE 2:** Comparing the average change of biometric factors between different groups according to ANOVA with LSD test.

Dependent variable			Mean difference (I–J)	SE	Significance	95% Confidence interval	
						Lower bound	Upper bound
Complete thickness (μm)	SVF	PRP	104.50000*	28.54066	**0.001** *	46.4336	162.5664
		PLACEBO	112.83333*	28.54066	**0.000** *	54.7669	170.8997
	PRP	SVF	−104.50000*	28.54066	**0.001** *	−162.5664	−46.4336
		PLACEBO	8.33333	28.54066	0.772	−49.7331	66.3997
	PLACEBO	SVF	−112.83333*	28.54066	**0.000** *	−170.8997	−54.7669
		PRP	−8.33333	28.54066	0.772	−66.3997	49.7331
Dermal thickness (μm)	SVF	PRP	98.75000*	29.01737	**0.002** *	39.7137	157.7863
		PLACEBO	107.50000*	29.01737	**0.001** *	48.4637	166.5363
	PRP	SVF	−98.75000*	29.01737	**0.002** *	−157.7863	−39.7137
		PLACEBO	8.75000	29.01737	0.765	−50.2863	67.7863
	PLACEBO	SVF	−107.50000*	29.01737	**0.001** *	−166.5363	−48.4637
		PRP	−8.75000	29.01737	0.765	−67.7863	50.2863
Complete density	SVF	PRP	1.09000	1.52576	0.480	−2.0142	4.1942
		PLACEBO	3.37833*	1.52576	**0.034** *	0.2742	6.4825
	PRP	SVF	−1.09000	1.52576	0.480	−4.1942	2.0142
		PLACEBO	2.28833	1.52576	0.143	−0.8158	5.3925
	PLACEBO	SVF	−3.37833*	1.52576	**0.034** *	−6.4825	−0.2742
		PRP	−2.28833	1.52576	0.143	−5.3925	0.8158
Epidermal density	SVF	PRP	2.77000	1.87061	0.148	−1.0358	6.5758
		PLACEBO	4.55083*	1.87061	**0**.**021** *	0.7451	8.3566
	PRP	SVF	−2.77000	1.87061	0.148	−6.5758	1.0358
		PLACEBO	1.78083	1.87061	0.348	−2.0249	5.5866
	PLACEBO	SVF	−4.55083*	1.87061	**0.021** *	−8.3566	−0.7451
		PRP	−1.78083	1.87061	0.348	−5.5866	2.0249
Dermal density	SVF	PRP	1.05333	1.59082	0.512	−2.1832	4.2899
		PLACEBO	3.70333*	1.59082	**0.026** *	0.4668	6.9399
	PRP	SVF	−1.05333	1.59082	0.512	−4.2899	2.1832
		PLACEBO	2.65000	1.59082	0.105	−0.5866	5.8866
	PLACEBO	SVF	−3.70333*	1.59082	**0.026** *	−6.9399	−0.4668
		PRP	−2.65000	1.59082	0.105	−5.8866	0.5866
Patient satisfaction	SVF	PRP	1.16667*	0.26272	**0.000** *	0.6322	1.7012
		PLACEBO	2.66667*	0.26272	**0.000** *	2.1322	3.2012
	PRP	SVF	−1.16667*	0.26272	**0.000** *	−1.7012	−0.6322
		PLACEBO	1.50000*	0.26272	**0.000** *	0.9655	2.0345
	PLACEBO	SVF	−2.66667*	0.26272	**0.000** *	−3.2012	−2.1322
		PRP	−1.50000*	0.26272	**0.000** *	−2.0345	−0.9655
Physician satisfaction	SVF	PRP	1.16667*	0.23925	**0.000** *	0.6799	1.6534
		PLACEBO	2.50000*	0.23925	**0.000** *	2.0132	2.9868
	PRP	SVF	−1.16667*	0.23925	**0.000** *	−1.6534	−0.6799
		PLACEBO	1.33333*	0.23925	**0.000** *	0.8466	1.8201
	PLACEBO	SVF	−2.50000*	0.23925	**0.000** *	−2.9868	−2.0132
		PRP	−1.33333*	0.23925	**0.000** *	−1.8201	−0.8466

* The mean difference is significant at the 0.05 level.

*Note:* Bold values are statistically significant.

### 3.4 Physician's and Patients' Satisfaction

**FIGURE 2:**
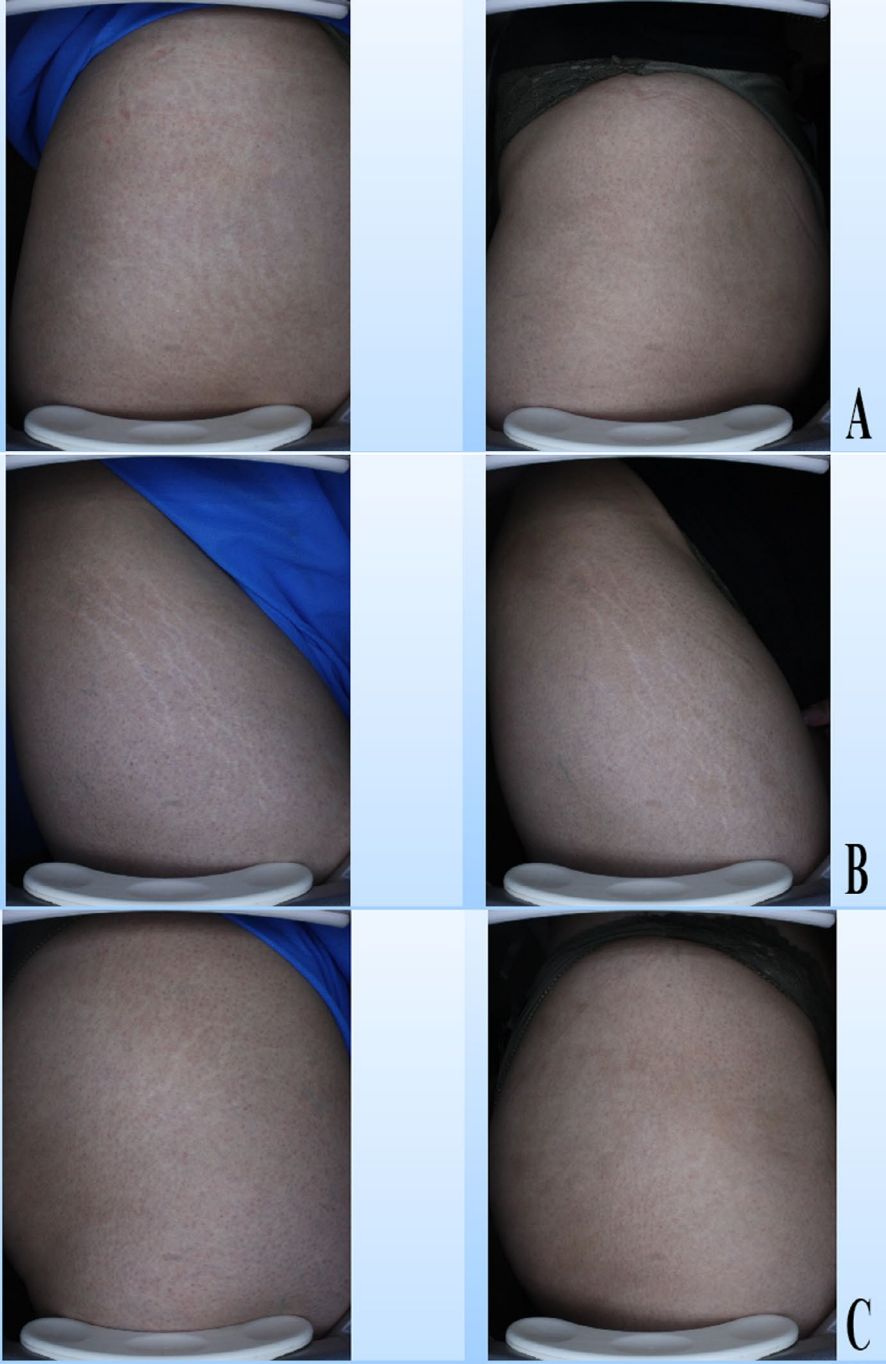
(Case 1). Clinical images of the three intervention groups studied, before (left) and after (right), (A) Er:YAG laser alone, (B) Er:YAG laser+SVF, (C) Er:YAG laser+PRP.

**FIGURE 3:**
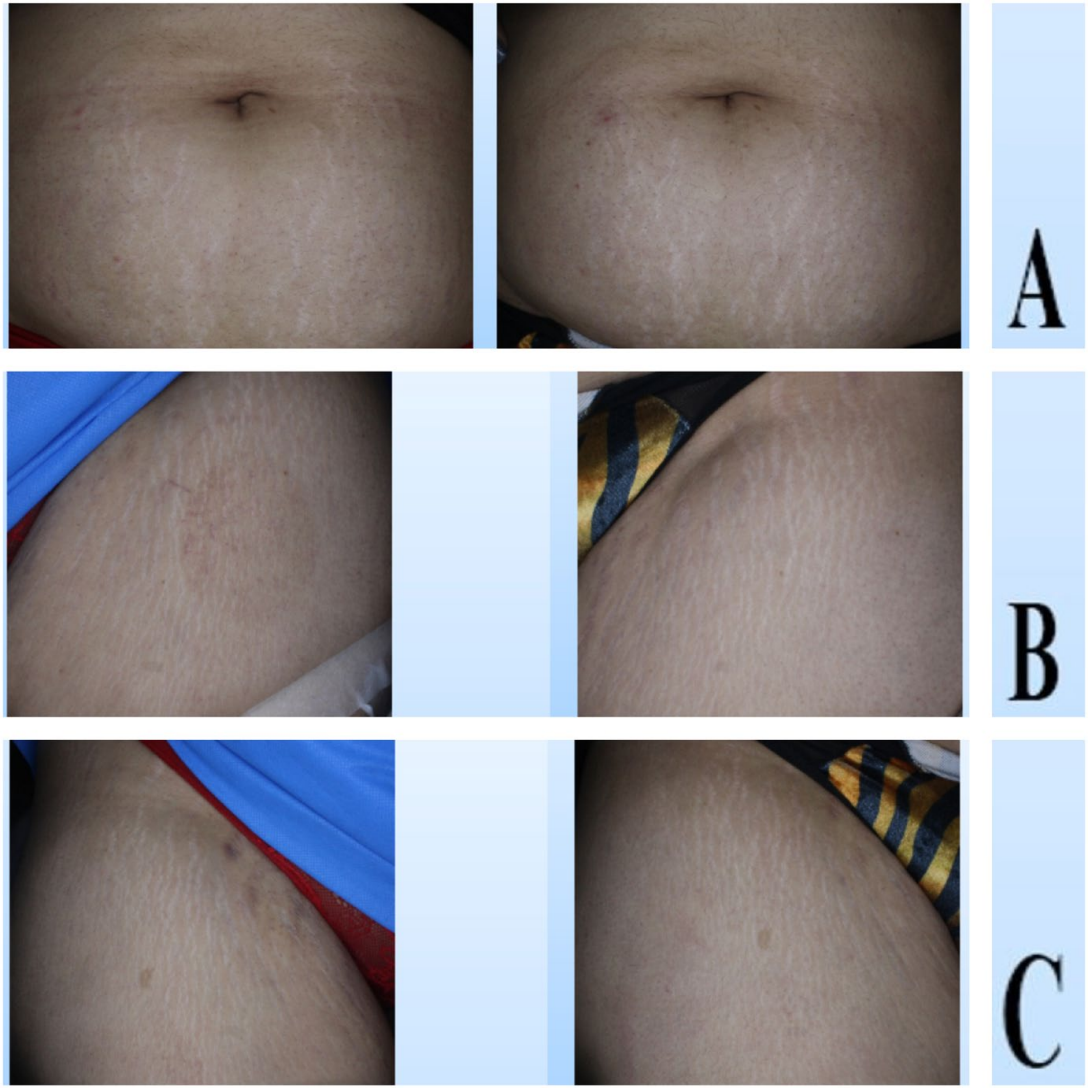
(Case 2). Clinical images of the three intervention groups studied, before (left) and after (right), (A) Er:YAG laser alone, (B) Er:YAG laser+SVF, (C) Er:YAG laser+PRP.

In the comparison of patient satisfaction scores between the three studied groups, the mean satisfaction score of patients in the SVF group was significantly higher than the PRP and placebo groups, Figures 2A–C and 3A–C (*p*: 0.001).

Also, the average satisfaction score of physician's satisfaction in the SVF group was significantly higher than the PRP and placebo groups (*p*: 0.001) (Table 3). No significant posttreatment complications were observed in this study.

**TABLE 3:** Comparing the physician's and patients' satisfaction between different groups.

Factor	Group	Mean	SD	95% CI		*p*
Patients' satisfaction	SVF	4.67	0.49	4.35	4.98	**0.001**
	PRP	3.50	0.80	2.99	4.01	
	PLACEBO	2.00	0.60	1.62	2.38	
	Total	3.39	1.27	2.96	3.82	
Physician's satisfaction	SVF	4.67	0.49	4.35	4.98	**0.001**
	PRP	3.50	0.52	3.17	3.83	
	PLACEBO	2.17	0.72	1.71	2.62	
	Total	3.44	1.18	3.04	3.84	

*Note:* Bold values are statistically significant.

## 4 Discussion

Striae distensae can have a destructive effect on people's physical appearance, beauty, and subsequently on their psychological state [27]. Considering the importance of investigating new treatment methods, this study discussed the therapeutic effect of Er:YAG laser alone and in combination with SVF and PRP.

Several studies described the use of Er:YAG laser for SD [10, 11, 12, 13, 14, 28]. In a case report by Gauglitz et al. [14], the efficacy of Er:YAG laser was much higher than PDL, regarding the treatment of SD at an early stage. The authors found the 2940‐nm Erbium:YAG fractional laser to improve texture and color of SD, after 3–5 treatment sessions, in two cases of axillary SD [14]. Another study [12] concluded that a variable square pulse Er:YAG laser resurfacing is a promising treatment for SD. A common side effect was transient postinflammatory hyperpigmentation (PIH), particularly in patients with darker skin, lasting up to 6 months [12].

According to Shen et al. study bipolar fractional radiofrequency (FRF) is as effective and safe as the 2940‐nm Er:YAG laser in SD treatment [13]. Wang and Song [29] combined Erbium fractional laser with intense pulsed light (IPL) to treat striae gravidarum (SG) in 60 patients in three sessions with 4‐week intervals. Results showed a significant reduction in the area of stretch marks from 7.89 ± 0.49 cm^2^ to 4.94 ± 1.16 cm^2^, improved elasticity and thickness, and decreased width and grayness of the marks (*p* < 0.001 for all). Patient satisfaction was high at 96.67% [29]. In concordance with the established efficacy of Er:YAG laser in SD treatment, all groups in this study achieved some response to the treatment, based on the biometric and objective and subjective evaluation. However, we are searching for a therapeutic option that provides faster results and higher satisfaction.

It is important to note that a recent systematic review found no significant difference in the effectiveness of ablative lasers, nonablative lasers, radiofrequency, and intense pulsed light in reducing the width of striae distensae, with all showing effectiveness [30]. However, it is also worth mentioning that the Er:YAG laser is associated with fewer thermal damage‐related side effects, which is particularly important for individuals with darker skin tones. Another systematic review indicated that microneedling is more effective than laser treatment for the treatment of striae distensae (*p* = 0.04), with a lower incidence of postinflammatory hyperpigmentation (PIH) (*p* = 0.0003). However, it is associated with significant higher levels of pain (*p* < 0.00001) [31]. It is recommended that future clinical trials focus on comparing microneedling and laser treatments for this condition.

The results of this study showed that the average change in biometric factors between the different groups was better in the SVF group than in the other groups. Thus, the average indices of complete thickness, dermal thickness, complete density, epidermal density, and dermal density after the intervention compared with before the intervention were significantly higher in the SVF group than in the other groups. Previous studies have shown the effect of SVF on improving skin texture [17, 32]. Transplantation of SVF cells can accelerate the regenerative power of the skin caused by mechanical stress and cause an increase in clinical tissue regeneration [17]. The results of Tan et al. [32] during a 2‐year follow‐up period showed that SVF induced skin regeneration and improved mechanical stretch. However, a systematic review found that most common treatment modalities are equally effective in treating SD and do not differ significantly in terms of side effects and treatment duration. In the combined treatment category, the combination of two or more methods is usually better than the use of any single method [33]. In concordance with the mentioned studies, the results of the combination groups were more significant compared with the placebo group.

The efficacy of the SVF method in the treatment of other skin lesions has been demonstrated. Lee et al. [34] documented a significant increase in scar tissue scores in the SVF group and patients had better outcomes. In another study comparing fat grafting and SVF grafting for skin improvement, VISIA scores showed that survival of SVF‐enriched fat grafts had significantly higher efficacy [35]. In another study, SVF injection was effective in treating horizontal neck wrinkles, and histological evaluation showed that SVF gel increased the collagen density of neck wrinkles [36]. In a 2019 study by Ou et al. [37] that investigated the effect of SVF‐GEL in the treatment of scars, SVF‐GEL transplanted patients survived 6 months after surgery without complications, and the degree of scar depression was significantly improved with lighter pigmentation and softer texture compared with before surgery, and the scores of the validated scar scale (VSS) were also significantly improved in terms of color and flexibility. Similarly, current study demonstrated notable improvement regarding ultrasonic indices of skin only after one session of combination therapy of SVF and fractional laser.

In the present study comparing physician and patient satisfaction, it was found that mean satisfaction was significantly higher in the SVF group than in the PRP and placebo groups. Regarding patient satisfaction, the SVF method was acceptable in recent studies [35, 36]. In a survey of Striae patients, satisfaction with treatment with Er:YAG laser combined with spatially modulated ablation (SMA) was acceptable [28].

In our study, the combination of Er:YAG laser and PRP was effective in treating SD. The results of a systematic review showed that treatment with PRP resulted in an increase in epidermis thickness, grooves, and collagen/elastin formation [38]. Similarly, the improvement in thickness of dermis and epidermis and density of dermis was significant in current study. In one study, the degree of improvement of SD with CO_2_ laser was found to be mild in two‐thirds of patients, 46.7% had a moderate response, and 20% of patients had unchanged clinical status [39].

A recent systematic review [9] revealed that fractional lasers are the most commonly used method for treating striae distensae (SD). However, PRP demonstrated a higher complete response rate (4% for fractional lasers vs. 7% for PRP). Another noteworthy finding was that response rate may be associated with factors such as the patient's skin type, age, and the duration of SD. Combining CO₂ laser treatment with PRP injections achieved a significantly higher complete response rate, (17%) compared to either modality used alone. It is important to note that our study was conducted on Caucasian patients with a mean age of 39.16 years, Fitzpatrick skin Types II–IV, and long‐standing SD lesions. In our study, we suggest combining the Er:YAG laser, which has a lower incidence of PIH and higher effectiveness, with stromal vascular fraction (SVF) therapy as an alternative to PRP.

The present study showed that SVF injection was acceptable in terms of biometric factors and treatment satisfaction. Our results also showed adequate safety in relation to the treatment methods performed, such that no significant complications were observed after the treatments. The safety of this method has also been demonstrated in previous studies [16, 17]. According to another finding, injection of SVF from adipose tissue resulted in better healing of burn wounds, and this method had no particular side effects [8]. In another study, scar healing of 63% and 65% was observed after 1 year in patients treated with SVF and the combination of PRP and nanofat, respectively. All patients expressed satisfaction with the texture, softness, and contour of the treated area. MRI scans showed no signs of cyst formation or microcalcification. There were no complications reported, and the results were durable, with a mean follow‐up period of 60 months, indicating that SVF is a safe and effective intervention [40]. It should be noted that while a single session of SVF combined with fractional laser yields satisfactory results, the SVF procedure is both time‐consuming and expensive. Therefore, it may be most appropriate for patients who have not responded adequately to other treatments.

A recent study by Behrangi et al. [41] demonstrated that combining microneedling with mesenchymal stem cell‐derived conditioned medium significantly improves the treatment of SD, compared to microneedling with saline over three sessions at 3‐week intervals, based on subjective assessment. Further research is needed to compare these innovative treatment options for SD in order to determine the most effective approach.

One of the strengths of this study was the use of biometric variables. The lack of a similar study using biometric indices in the treatment of SD was a limitation of this study that made it difficult to compare the results. However, it is recommended that studies with a larger sample size be conducted to confirm the results.

## 5 Conclusion

Based on the results of this study, the combination therapy with Er:YAG laser is superior to the Er:YAG laser with placebo. The SVF and Er:YAG laser combination achieved more satisfactory results compared to the PRP and Er:YAG laser combination, as evidenced by biometric data and patient satisfaction assessments. SVF injection plus Er:YAG laser can be considered an effective and safe treatment modality for striae distensae. Further studies with longer follow‐up periods and larger sample sizes are recommended to thoroughly evaluate the advantages and disadvantages of this method.

## Author Contributions

Contributions to the current study include M.R., Z.M., A.D., M.N., and M.A.N. in the study idea and design, literature review, and drafting and revising the manuscript critically for important intellectual content. S.Z., A.G., E.B., and M.A.N. were involved in conducting the trial, data gathering, drafting the proposal, obtaining ethical committee approval, and revising the manuscript critically for important intellectual content. M.R., Z.M., A.G., M.N., Z.E., and E.B. participated in the literature review, analysis, and interpretation of the revised version, and drafting the manuscript. A.G. and E.B. contributed to the proposal preparation, statistical analysis, and drafting the revised manuscript. M.A.N. and E.B. supervised the study, gathered data, conducted literature reviews, and drafted the manuscript, and both serve as corresponding authors. Finally, R.Z. revised the manuscript according to reviewers' comments. All authors have read and approved the final version to be published and agreed to be accountable for all aspects of the work.

## Ethics Statement

All information collected was kept confidential and evaluated without a specific name. Participants in this project adhered to all Helsinki ethical principles (Name of the registry: Iranian Registry of Clinical Trials, IRCT registration number: IRCT20220322054341N2, URL: https://www.irct.ir/search/result?query=IRCT20220322054341N2; registration date: 2023‐01‐20). This research was approved by the Research Council under the ethics code number IR.IUMS.FMD.REC.1401.510.

## Consent

The authors obtained written consent from the patients for the publication of their photographs.

## Conflicts of Interest

The authors declare no conflicts of interest.

## Data Availability

The data that support the findings of this study are available from the corresponding author, upon reasonable request.
